# Universal CAR 2.0 to overcome current limitations in CAR therapy

**DOI:** 10.3389/fimmu.2024.1383894

**Published:** 2024-06-19

**Authors:** Lara Sophie Schlegel, Coralie Werbrouck, Michael Boettcher, Patrick Schlegel

**Affiliations:** ^1^ School of Medical Sciences, Faculty of Medicine and Health, University of Sydney, Sydney, NSW, Australia; ^2^ Department of Pediatric Surgery, University Medical Centre Mannheim, University of Heidelberg, Heidelberg, Germany; ^3^ Department of Pediatric Hematology and Oncology, Westmead Children’s Hospital, Sydney, NSW, Australia

**Keywords:** CAR (chimeric antigen receptor) T-cell therapy, antibody therapies, iPSC (induced pluripotent stem cell), cancer immune cell therapy, individualized cancer therapy

## Abstract

Chimeric antigen receptor (CAR) T cell therapy has effectively complemented the treatment of advanced relapsed and refractory hematological cancers. The remarkable achievements of CD19- and BCMA-CAR T therapies have raised high expectations within the fields of hematology and oncology. These groundbreaking successes are propelling a collective aspiration to extend the reach of CAR therapies beyond B-lineage malignancies. Advanced CAR technologies have created a momentum to surmount the limitations of conventional CAR concepts. Most importantly, innovations that enable combinatorial targeting to address target antigen heterogeneity, using versatile adapter CAR concepts in conjunction with recent transformative next-generation CAR design, offer the promise to overcome both the bottleneck associated with CAR manufacturing and patient-individualized treatment regimens. In this comprehensive review, we delineate the fundamental prerequisites, navigate through pivotal challenges, and elucidate strategic approaches, all aimed at paving the way for the future establishment of multitargeted immunotherapies using universal CAR technologies.

## Introduction

Immune oncology has experienced a major breakthrough with the development of CAR expressing immune effector cells (1). CAR receptors are synthetic immune receptors comprised of cell-specific functional protein units strategically assembled to create dimeric receptors which facilitate a multifaceted response upon engagement of the CAR with its targeted molecular structure (2). Other than the recognition domain that can be derived from different species, the functional subunits of a CAR are generally derived from human proteins. Each module displays unique properties that mediate specifically defined functions. The CAR is comprised of an ectodomain, which consists of an antigen-recognition domain and a spacer, a transmembrane domain and the intracellular signaling unit that can be composed of one or multiple signaling domains (3).

## CAR design

In most instances, the extracellular signaling domain consists of a murine antibody-derived single chain variable fragment (scFv) (4) or a camelid heavy chain variable (VHH) (5) but may also be derived from fully human VHH (6), scFv or ligand (7) for the targeting of overexpressed cognate receptors in cancer (APRIL – BCMA & TACI) (8), other species’ antibody variable chains, or alternatively, artificial recognition domains, such as D-domains (9). To reduce the immunogenicity, humanized murine single chain variable fragment (scFv) (10) or deimmunized camelid VHH may be used (11).

**Figure 1:**
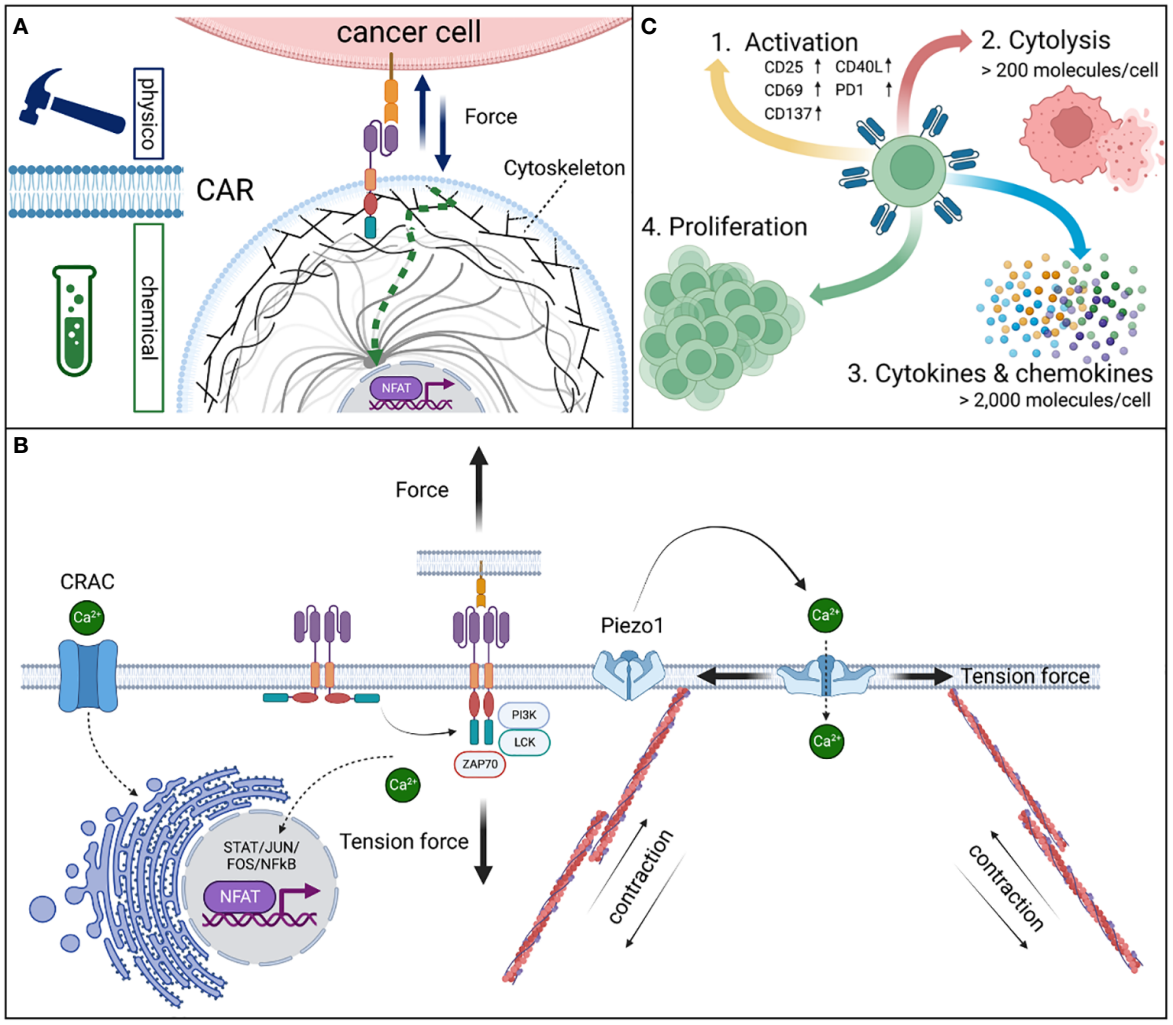
Physicochemical signal conversion of CAR receptors. CARs are **(A)** mechanoreceptors that allow for the transmission of physical tension force to convert into complex chemical signals from the outside of the cell into changing the activation state and downstream functions of the cell including immediate responses, such as the formation of a cytolytic synapse and migration or intermediate and long-term adaptions by changes in the gene expression. **(B)** The mechanical force on CAR expressing effector cells is mediated by the binding interaction of the CAR recognition domain and the antigen leading to a conformational change of the cytoplasmic signaling domains. These steric adaptions result in the accessibility of phosphorylation sites and the cognate alignment of signaling proteins that initiate a downstream signaling cascade which is based on enzymatic phosphorylation steps of subsequent signaling proteins in a defined order (13). Calcium serves as an important second messenger in the signaling process. Piezo1 calcium channels are opened by tension forces to the cell membrane by active deformation of the cytoskeleton via actin filaments, allowing calcium influx into the cytoplasm. Especially, during the formation of the cytolytic synapse Piezo1 dependent calcium influx is required (14). Further, cellular calcium metabolism is tightly regulated by the endoplasmic reticulum (ER). The ER serves as a large calcium storage filled with calcium by the calcium release activated channel (CRAC) (15). Upon calcium release from the ER and binding thereof to calcineurin, a phosphatase, is activated. Calcineurin serves as a key modulator of the transcription factor “nuclear factor of activated T cells” (NFAT) and thus serves as a key modulator of T cells in general (16). Dephosphorylation of the nucleus localization signal allows the translocation of NFAT into the nucleus and induce selective gene expression. Dysfunctional CRAC prevents the development of T cells and is the cause of the severe combined immunodeficiency (SCID), a life-threatening inborn immune deficiency (17). **(C)** The efficient transmission of the physicochemical signal leads to various effector functions in CAR expressing effector cells. In a resting CAR, 1^st^ cell activation by the CAR receptor is detectable by the expression of activation markers. 2^nd^ Cytolytic activity in high affinity CARs (KD < 1 nM) is induced at an antigen density of as low as >200 molecules per cell, whereas significant changes to the 3^rd^ gene expression, cytokine and chemokine secretion require >2,000 molecules per cell (18). Thus, 4^th^ a strong triple signal of ^i)^ CAR-mediated antigen recognition (CD3ζ), ^ii)^ co-stimulation (CD28), and ^iii)^ cytokine support (IL2) are required to induce proliferation.

The spacer is essential in the configuration of the CAR, as its length determines the binding proximity and prevents steric hindrances, hence facilitating access to the epitope. The transmembrane domain anchors and stabilizes the receptor in the cell membrane as well as connecting the extracellular components with the intracellular signaling compartment referred to as the endodomain that contains one or several costimulatory and/or signaling domains (3). The specific modules of the distinct CAR define the functional properties of the synthetic immune receptor (2). These are mainly the response and proliferation kinetics of the CAR expressing cell that are dependent on the signal transduction from the extracellular domain to the intracellular signaling domains (12). Therefore, the deliberate combination of functional protein units allows for optimized and requirement-adjusted functionality. The basic understanding and principles of the CAR introduced as a physicochemical receptor and its measurable effector functions are illustrated and explained in detailed in **Figure 1**.

## Universal CAR approaches

Universality in CAR T cell therapy can refer to both 1^st^ the universal applicability of a CAR T cell product with no recipient restrictions due to T cell receptor/HLA incompatibility (19) and 2^nd^ the capability of targeting any antigen of interest facilitated by indirect CAR technologies via adapter molecules (20).

All US-FDA/EMA-approved CAR T products are manufactured from autologous patients’ T cells and have demonstrated high clinical efficacy (3). Per definition, autologous CAR T cell products cannot induce GvHD or is subject to HLA-based immune rejection. Autologous CD19- and BCMA-targeted CAR T cells robustly engraft in most patients (21). The CARs’ architecture define their biology, metabolism and persistence (4, 22, 23), which generally is not subject to primary immunological CAR rejection, however, the induction of humoral anti-CAR immunity may limit the engraftment and success of a second CAR T cell application in due course (24).

To date most CAR T cell therapies are based on autologous products which implies the donor and recipient to be the same individual (23). Posttransplant manufactured autologous CAR T cell products are considered autologous even if the genetic origin of the hematopoietic system is donor-derived (25) rather than self-derived. For example, a CAR T cell product created post-transplant can come from an HLA-matched donor or even a haploidentical donor with an HLA mismatch. The cell source can be either the recipient (patient) or the same donor used for the hematopoietic allogeneic stem cell transplantation (HSCT) (26). In general, HLA-matched and haploidentical allogeneic CAR T cells both require to be administered post transplantation. Otherwise, the CAR T cell product is rejected by the recipient’s immune system (27).

The major advantage of third-party allogeneic HLA-mismatched donor-derived immune effector cells, besides manufacturing costs, is the immediate availability of these products at any point in time (28). The manufacturing process of autologous CAR T is comprised of leukapheresis, T-cell isolation and stimulation, gene delivery, *ex vivo* expansion, cryopreservation and quality controls (29). The logistics in the complex multi-step preparation cells is prone to errors. Usually, time-consuming testing of T cells prior to manufacturing is required. Further, the therapeutic success of autologous CAR T cells may be constraint by the quantity and quality of the isolated peripheral blood cells of the patient (30).

The idea of third-party CAR T cells is driven by the success of third-party virus-specific T cells (ADV, CMV, EBV) used in the posttransplant setting to treat viral reactivation, as these allogeneic cells can transfer transient antiviral immunity to bridge the time until the patients’ T cells have reconstituted and provide viral immune protection (31, 32). In patients eligible for CAR T cell therapy, time is of the essence and one key critical determining factor of survival (33). Additionally, the risk for manufacturing failure in CAR T cell therapy varies significantly (>10%) depending on the pre-treatment history and the primary underlying cancer (34, 35). Further, the clinical efficiency of a bridging chemotherapy to enable the patient to reach the CAR T cell treatment a stable clinical state to receive the CAR T cell product (36). Thus, manufacturing failure can compromise the patients’ opportunity for a successful administration of CAR T cells (34, 37). As such, third-party allogeneic CAR T cell therapy provide a bridge to transplant for selected patients (38).

**Figure 2:**
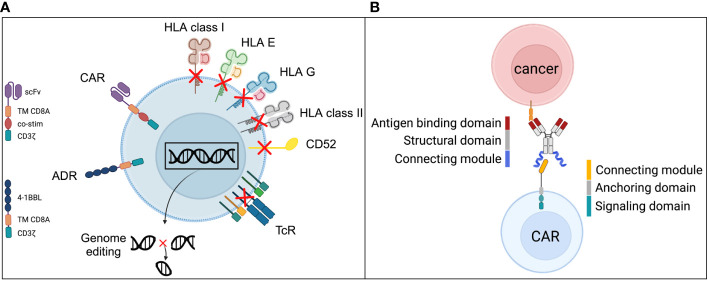
Universal CAR strategies have two meanings. Universal “allogeneic CARs” generated from iPSCs require a series of genetic alterations to enable their successful and safe clinical use. Current concepts **(A)** include modifications that reduce the likelihood of allorejection by gene disruption of ß2-microglobulin to abrogate the expression of HLA class I structures as well as by gene disruption of the class II MHC transactivator (CIITA) to abrogate HLA II expression (41, 42). To exclude iPSC-derived cells from harm through alemtuzumab, the CAMPATH-1 antigen CD52 is required to be knocked out as it has been done for third-party allogeneic CAR products (43). Alemtuzumab can then be used in the CAR preparative regimen and facilitate longer engraftments of the allogeneic cells. However, alemtuzumab induces long-lasting severe cellular and humoral immune deficiency which attracts serious infectious complications (44). In order to reduce the risk for graft-versus-host disease, the T cell receptor (TcR) expression has to be disrupted and is usually achieved by the genetic knockout of the constant alpha (TRAC) chain of the TcR. Since the TcR is the responsible receptor for alloreactivity in GvHD direction, the TcR-KO is the most effective strategy to silence the primary T cell function (42). Novel strategies to increase the resistance to potential allorejection was introduced by Mo et al in 2021 via an alloimmune defense receptor (ADR) (45). It’s a ligand based (4-1BBL) signal converting receptor by providing a CD3ζ signaling if the allogeneic iPSC derived CAR expressing cell gets in contact with activated immune cells, such as T cells and NK cells that express the co-stimulatory receptor 4-1BB (CD137). The CD3ζ signal increases the resistance to allorejection mechanisms induced by T cells and NK cells. **(B)** The basic principle of adapter CARs is the indirect targeting of the CAR expressing cells via advanced antibody-dependent cellular cytotoxicity (ADCC). Since the adapter molecule, e.g., an antibody is interchangeable the specificity of the targeting is theoretically unlimited. The structure of the adapter molecule is comprised of three domains with distinct functions. The antigen binding domain corresponds to the primary antibody binding capability to a structure expressed on target cells. The structural domain provides stability to the molecule and supports manufacturability, the connecting module interacts with the CAR receptor and facilitates the highly specific recognition of the adapter molecule only by the CAR expressing cell and no other immune cells. The counterpart is the CAR expressing cell and especially the CAR receptor itself comprised of the cognate connecting module to allow the highly specific recognition and interaction with the adapter molecule (20). Further, the anchoring domain stabilizes the receptor in the cell membrane and the signaling domain provides the cell with the downstream signaling to ignite cellular functions according to the design of the receptor.

Partially HLA-matched third-party virus-specific T cells have been documented to contribute controlling virus reactivation posttransplant in immunocompromised patients (39). In order to guarantee anticancer activity by universal third-party CAR T cells, patients undergo a preparative regimen including the treatment with alemtuzumab (IgG1_K_, t_1/2_ = 6-21 days) targeted to CD52 which leads to a profound and enduring depletion of B- and T lymphocytes, NK cells and monocytes (40). Genetic modifications via CRISPR/Cas9 offer to disrupt the TRAC locus and CD52 genes, indicated in **Figure 2**. These genetic alterations prevent the immediate rejection of the HLA mismatched effector cells via the depletion of the recipients’ lymphocytic compartment by alemtuzumab and the inactivation of the TRAC leads to a collapse of the TcR preventing the induction of graft versus host disease (GvHD) by third-party T cells in the immunocompromised host (21, 46). A CD19-targeted allogeneic product was shown to be safe and efficacious in heavily pre-treated adult patients with relapsed and refractory B-ALL with allogeneic off-the-shelf UCART19 cells in the clinical trial [CALM] (NCT02746952) (21). Alternative cell sources for third-party off-the-shelf CAR products include NK cells isolated from cord blood (47), γδ T cells collected from healthy donors (48), or the leukemic cell line NK-92 (49, 50). NK cells and γδ T cells do not induce GvHD but are subject to rejection by the recipient’s immune system in the allogeneic setting and thus can only be used transiently post lymphodepleting chemotherapy (51).

Another approach that is proving to be viable involves using induced pluripotent stem cells (iPSCs) as the primary cell source for the cellular CAR product. iPSC-derived CAR T are at early stage of development. The main principles are illustrated and outlined in **Figure 2A**. iPSCs are used to create in silico designed, genetically tailored, highly defined and characterized cellular products according to unique functional and immunological requirements (52).

Genetic modifications are achieved using various gene engineering tools tailored for specific applications. The historical development, feasibility, scalability, advantages, and disadvantages of these tools are comprehensively discussed by Adli et al. in the review article “The CRISPR Toolkit for Genome Editing and Beyond” (53). Today, the key tools include CRISPR/Cas9, TALENs, ZFNs, homologous recombination, the PiggyBac transposon system, and the Sleeping Beauty transposon system, among others. Delivery systems for these genome editing tools include electroporation (54, 55), transfection with chemical compounds (56), AAV-based methods (57), and retroviral/lentiviral gene delivery (58). We reference primary literature and review articles that delve into the detailed aspects of these tools, such as “Genome Editing with CRISPR–Cas Nucleases, Base Editors, Transposases, and Prime Editors” by Anzalone et al (59).

Theoretically, iPSC-derived cellular products can grow endlessly and multiply countlessly in numbers, thus providing enough cells for third-party CAR T products from one single original donor source with no limits (28). Because the artificial iPSC-derived CAR T cells are HLA mismatched, they require at the minimum the same genetic modifications as “conventional” third-party CAR T cell products to reduce alloreactivity and graft rejection. However, counter to third-party allogeneic CAR T cells, there are no constraints in the genetic engineering as iPSC can be cultured infinitively (60). Of note, even though iPSC-derived cells can be well-characterized and extensively tested genetically and functionally, they bear a potential risk for mutations, genetic disruptions and rearrangements of functional genes (61) which could lead to secondary hard-to-treat iPSC-CAR-leukemia with resistance to treatment which has been observed in autologous CD19CAR T cells with piggyBac transposon-based gene delivery (62). In addition, decreased amplification and shorter persistence of iPSC-based CAR products *in vivo* in preclinical models required additional modifications to ensure robust anticancer activity (28). While iPSC-derived CAR T-cell therapy holds great promise for personalized cancer treatment, many challenges remain to ensure a safe and effective clinical translation (63).

The term “Universal CAR T” is also referred to technologies that enable CAR receptor expressing immune effector cells to engage with an unlimited variety of different antigens (64). This can be achieved with adapter CAR T platform technologies thoroughly discussed by Arndt (65) et al as well as Liu (66) et al. The basic principle of adapter CAR T cells is illustrated in **Figure 2B**. The antigen recognition and CAR signaling is decoupled and requires the correct assembly of three components 1^st^ the adapter CAR expressing cell with their surface-expressed CAR receptors, 2^nd^ the adapter molecule and 3^rd^ the target antigen expressing cell (20). The recognition domain of adapter CARs is either targeted to a non-human “neo”-epitope (20, 67) non-existent in the human body or an inaccessible, e.g., intracellular (68) structure for the CAR recognition domain, but instead is only found as a targetable moiety on adapter molecules (65). In consequence, adapter CARs are functionally inert cells that only engage with target cells via the CAR receptor in the presence of the corresponding adapter molecule (65). Additionally, redirecting conventional CARs has also been employed to broaden the spectrum of targeting (69, 70). The specificity of the adapter molecule is interchangeable and thus the number of targetable antigens can be extended by introducing additional adapter molecules into the system (20, 71). Analogue to antibody-dependent cellular cytotoxicity (ADCC) with NK cells (72), adapter CAR technologies facilitate an artificially potentiated type of ADCC that allows for the tight regulation of effector functions (67), transient targeting, combinatorial synchronous multitargeting and sequential targeting (20).

An overview of adapter CAR systems is provided in **Table 1** in alignment with **Figure 2B**. Contemporary translational aspects are summarized in the section - Clinical experience with adapter CAR technologies.

**Table 1:** Adapter CAR systems.

Connecting module CAR	Connecting module adapter molecule	References
Antibody structure		
CD16/CD32A	Fc domain IgG1	(73)
scFv (P329G substitution)	Fc domain P329G	(74)
Chemical conjugation		
scFv (FITC)	fluorescein isothiocyanate (FITC)	(75)
monomeric streptavidin 2 (mSA2)	EZ-link NHS biotin	(76)
dimeric avidin	EZ-link NHS biotin	(77)
scFv (LLE)	EZ-link NHS-LC-LC-biotin (LLE)	(20)
Peptide tag		
scFv (La)	La/SS-B	(68, 78)
scFv (GNC4)	GNC4 transcription factor	(67, 79)
d-domain	neo-peptide	(80)
Protein tag		
scFv (CD19)	CD19 fusion protein	(70)
Dimerizing domains		
leucin zipper	leucin zipper	(81)
Covalent binding		
SpyCatcher	SpyTag	(82)
DNA methyltransferase (MGMT)	O^6^-benzylguanine (BG)	(83)
Bispecific antibody		
folat receptor alpha (FRα)	bispecific antibody (FRα x target antigen)	(84)
EGFRvIII	bispecific antibody (EGFRvIII x target antigen)	(85)
scFv	bispecific antibody (scFv G_4_S x target antigen)	(69)

The table summarizes technologies based on the modular CAR concept, where artificial immune receptors on CAR effector cells are redirected to any target antigen of interest using connecting molecules.

In a synergistic approach, iPSC derived third-party adapter CAR T cells may combine the features of iPSC-derived CAR immune effector cells (28) with the capabilities of versatile indirect CAR technologies – as *adapter CAR 2.0 (* 20).

## Basic requirements of CAR immune effector cells

### Baseline

Patients who are eligible for CAR T cell therapy, are deadly sick and have no further treatment options. FDA/EMA-approved CD19- and BCMA-specific CAR T cell products are mainly last line therapies, however, ironically provide high complete remission induction rates and increase overall survival in B-lineage cancers including multiple myeloma substantially (86). With excellent response rates in CD19 positive cancers, a relevant proportion of patients may experience a sustained complete response (which can be considered a cure from the primary disease) in 40% of B-ALL (4, 87) and DLBCL patients (88, 89), whereas in CLL the sustained complete response rate is 20% (90).

Despite the great success of CAR T cells, in multiple myeloma, the cancer cells’ antigen heterogeneity and immunosuppressive factors in the tumor niche have highlighted future challenges to be overcome (91). BCMA-specific CAR T cells have demonstrated significant clinical benefits in multiple myeloma patients that otherwise have no further treatment available to date (92). Yet, multiple myeloma is continuously and largely considered incurable and new treatment strategies are required to improve outcomes (93). Obviously, potent therapies justify being considered earlier in the treatment algorithm and with less than 10% of newly diagnosed multiple myeloma patients to receive CAR T cell therapy (94) because they die before being eligible according to international guidelines. In due course adjustments are required to be made carefully based on clinical outcomes. It is noteworthy that multiple myeloma patients who received the BCMA CAR T cell product idecabtagene vicleucel (bb2121) in the clinical trial KarMMa (NCT03361748) showed a median overall survival of 19.4 months (92) which represents a significant survival benefit compared to untreated patients, non-responders and patients with partial response only.

Despite significant responses in patients with various solid cancers treated with CAR T cells, such as disialoganglioside GD2 in neuroblastoma (95) and H3K27M-mutated diffuse midline gliomas (96), mesothelin (MSLN) in mesothelioma (97), claudin 18.2 (CLDN18.2) in pancreatic cancer (98), and IL13Rα2 in combination with EGFR in glioblastoma multiforme (99), CAR-based therapies for solid cancers face major limitations and challenges. Although patients may experience objective tumor regression and even complete remissions, most cancers inevitably relapse due to target antigen loss or antigen heterogeneity, which hinders primary complete responses (95, 97). Additionally, reduced CAR T cell persistence (96), the inability of T cells to infiltrate tumors, and the inhibition of CAR T-cell function by the immunosuppressive microenvironment (97) are significant barriers to durable responses. These issues can potentially be addressed by combinatorial strategies, such as integrating CAR T cells with immune checkpoint inhibition (100).

### CAR kinetics and sensitivity

Without immediate tumor control, patients may suffer from severe cancer-induced complications or die from cancer progression. Therefore, an immediate and effective anti-cancer response is essential for a successful CAR T cell therapy (4, 101). The containment of cancer growth is facilitated by the cellular cytotoxicity, the secretion of proinflammatory cytokines and the exponential proliferation, which are triggered by the antigen-specific CAR activation with rapid kinetics inducing effector functions in alignment with T cell receptor mediated immune responses (102). Especially, the trafficking and proliferation kinetics (103) to outnumber the cancer cells and the enduring anti-cancer response by the CAR T cells until the entirety of the cancer is cleared or reduced to a molecular residual disease are key requirements to prevent cancer recurrence (104). Additionally, the CAR must have a high sensitivity in terms of recognizing and eliminating low antigen density expressing tumor cells to ensure profound cancer control (12). Otherwise, the antigen low expressing cancer cells may escape the CAR-mediated recognition whereas the antigen negative cancer cell subsets cannot be recognized by the CAR T cells and in due course initiate the relapse (91, 105). Identifying the optimal CAR sensitivity and -kinetics depend on multiple factors, including the differential antigen expression in healthy versus cancerous tissue (106), the tumor microenvironment, the level of inflammation, and the responsiveness of the specific CAR design (12, 102). The activation state and exhaustion of CAR T cells are influenced by changing conditions during the anticancer response, including the cancer burden and the cytokine milieu (107, 108). Achieving a balance between initial and sustained response, tumor control, and managing both acute and chronic toxicity is crucial for effective therapy.

### Manifold effector functions

The basic primary effector functions of artificial immune cells like CAR T, CAR NK, chimeric TcR-like receptor and transgenic TcR expressing cells, are antigen-specific cellular cytotoxicity, cytokine production and proliferation (20, 57, 109, 110).

In reality, the immune effector cells are composed of a multitude of different cell subsets with various effector functions. The main αβT cell subsets are CD4^+^ T helper cells and CD8^+^ cytotoxic T cells. Yet, the various T cell subsets have been subclassified by the T cell receptor profile, the gene expression signature including decisive transcription factors, secretion of cytokines and chemokines as well as their cytokine- and chemokine receptor profiles which determine their responsiveness, adaptability and their specific functions (111–113).

While chemokine receptors and integrins determine the homing and preferential residence of T cells in certain tissues (114) the multifaceted effector functions including cytotoxicity and immunomodulation mediated by both CD4^+^ and CD8^+^ cells lead to their subclassification (115).

CD4^+^ αβT cells are classified as Th1, Th2, Tregs, TFH, TH22, TH17 and TH9. Cytokines range from proinflammatory IFNγ and TNFα released by Th1 cells to counterbalancing immunosuppressive cytokines IL10 and TGF-ß by Tregs (111). The interplay is important not only to prevent harm by hyperinflammation (116) but also to restore functions in order to maintain a potent immune response and circumvent terminal exhaustion of T cells (117).

CD8^+^ αβT cells are classified as Tc1, Tc2, Tc9, Tc17 and Tc22 and share a similar spectrum of cytokines like CD4^+^ cells ranging from proinflammatory to immunosuppressive functions as of IL12 and IL4, respectively (115). In relation to cancer entity the number and composition of tumor infiltrating lymphocytes (TILs), the frequency of CD8^+^ T cells and their subsets vary substantially (115). The prognostic value of TILs (CD4^+^ and CD8^+^) depends on the cancer type and the pathological TMN staging (118, 119).

Importantly, the maturation state of T cells defines their proliferative and regenerative capacity and is identified by the expression of cytokine- and chemokine receptors as well as activation and exhaustion markers (111).

Culturing conditions, including the cell culture media (e.g., glucose level) and supplements such as sera and cytokines (e.g., IL2, IL7, IL15, IL21, IL18), as well as cytokine dosing, initiation of T-cell activation and proliferation, and the duration of expansion, significantly impact the cellular composition of CAR products. These factors determine the maturation state and stemness of CAR effector cells, which in turn affect their proliferative capacity and clinical performance (120). The duration of CAR T cell expansion is crucial for supply, cost, and performance. CD19CAR-T tisagenlecleucel (CTL019), manufactured using the T-Charge^TM^ platform, is known as YTB323. This platform reduces manufacturing time from 9-10 days to less than 2 days. YTB323 has demonstrated that shortened manufacturing time preserves the stemness of CAR T cells, measured as the frequency of T_N_ and T_SCM_, and achieves the same tumor control as tisagenlecleucel at a 25-fold lower dose (121). Other strategies to induce stemness and enhance CAR T cell functionality include supplementing the culture media with inosine which induced profound metabolic reprogramming, from glycolysis to mitochondrial oxidative metabolism and the epigenome toward greater stemness. The same effect was induced by genetically modifying CAR T cells to overexpress adenosine deaminase (ADA-OE), an enzyme that metabolizes adenosine to inosine, thereby preventing inhibitory effects through the ATP, ADP, and AMP CD39/CD73 pathway (122). Additionally, overexpressing FOXO1, a transcription factor involved in regulating gluconeogenesis and glycogenolysis mediated by insulin, has been shown to enhance stemness, metabolic fitness, and CAR T cell performance (123).

Based on their immunomodulatory functions CD4^+^ and CD8^+^ cell subsets harmonize the immune response. Severe immediate or chronic overactivation of T cells can lead to detrimental effects and death observed in viral infections (124), autoimmunity, serotherapy during conditioning for allogeneic stem cell transplantation and CAR T cell therapy (125).

### Low immunogenicity

In all of the US-FDA approved CAR T cell products the extracellular recognition domain is either based on a murine scFv or a llama-derived VHH, hence contain non-human sequences, which may be identified by the immune system as foreign and induce the generation of CAR-rejecting antibodies (24) or T cell mediated rejection (126, 127). Both allorejection mechanisms can lead to the loss of the CAR T cell function or to the complete elimination of the CAR T cells (126). Since immunogenicity is impacted by molecular size, sequence dissimilarity and conformational structure, camelid VHH are in general considered to be less immunogenic. VHH are superior in chemical and physical properties including higher solubility, stability, smaller size (15kDa), they have a higher resemblance in sequence and conformational structure to human VHH compared to murine scFv (30kDa), and hence exhibit lower immunogenicity (128). Immunogenicity of non-human proteins can be reduced by humanization of murine scFvs and deimmunization of camelid VHH (24, 129). However, CAR T cells targeted to B-lineage associated antigens, such as CD19, CD20, CD22 and BCMA protect themselves from rejection as they deplete or interfere with the immune compartment that is responsible for the generation of anti-CAR antibodies (129).

## Limitations of CAR T

### The manufacturing process defines the product

CAR T cell products are defined by their original cell source and cell number. Unstimulated peripheral blood leukaphereses contain a median of 9.8x10^9^ total nucleated cells with 3.8x10^9^ total CD3^+^ cells (130). Thus, one of the most compromising aspects of CAR T cell generation is the misrepresentation of their entire primary heterogeneity in the whole T cell pool of an estimated 4x10^11^ number of cells (131), 1^st^ due to the low number of cells used for manufacturing [1x10^8^] with a median transduction efficiency of 46% (132) which corresponds to 0.01% (one ten thousandth) of the T cell pool, and 2^nd^ the collection from only one body compartment, the peripheral blood (130, 132). The heterogeneity of T cells in the peripheral blood and tissues are defined by differential functions dependent on the expression of certain integrins and chemokine receptors explaining the migration and residence tendency of the T cells in which they organize a complex interplay of the immune system (114).

Through the manufacturing process, various factors significantly impact CAR T characteristics. This process involves the drastic non-physiological antigen-independent activation of the T cell receptor complex (CD3) usually via the CD3 epsilon chain, often in combination with the co-activation of the CD28 costimulatory receptor (20). Additionally, continuous exposure to high concentrations of interleukin 7 (IL7), a T cell growth factor and regulator of Th1 and Th2 cytokine production (133) and/or the Th1-type cytokines, e.g., IL2 (120), IL15 (120, 134), IL21 (120) and/or the co-incubation with irradiated feeder cells (135), along with transgene delivery and the constitutive signaling and potential tonic signaling of the CAR receptor (136), collectively transform CAR expressing cells into artificial immune cells distinct from physiologic T and NK cells. Despite this distinction, CAR T cells share foundational properties with naturally matured and regrowing immune cells (137). Consequently, CAR expressing cells show similar behaviors and effector functions like natural immune cells but also exert additional functions and lack characteristics of their natural counterparts and have distinct metabolic signatures dependent on the CAR architecture (22). Signaling from T cell receptors and CAR receptors are fundamentally different even though CAR receptors mimic the TcR function and recruit the same downstream signaling proteins and engage with the same signaling pathways (138). Site-specific integration of CAR receptors into the TRAC locus facilitates the transgene expression according to the complex gene regulation of the TcR (57). The physiological CAR transgene expression has been shown to improve the CAR performance. Novel non-viral knock-in strategies may lower cost, complexity and time of CAR manufacturing. The technical challenge was to improve the delivery of homology-directed repair (HDR) templates of single-stranded DNA encoding the transgene instead of double-stranded DNA to reduce the toxicities in the electroporated cells (139).

These revolutionary gene editing tools have inspired the idea of site-specific integration of a novel immune receptor design mimicking the TcR. By exchanging the variable alpha and beta chains of the TcR with the antibody variable chains (VL and VH) of an antibody, the sensitivity of this advanced artificial immune receptor (HLA-independent TcR) was substantially increased to outperform classic CAR receptor design, demonstrated for antigen low expressing cancer cell line variants (NALM6_CD19Low_ and MOLM13_CD70Low_). Yet, the constitutive co-expression of CD80 and 4-1BBL was required to enhance persistence (110). Interestingly, after three decades of CAR research, the advancements of synthetic biology and gene engineering have revived the original idea of Zelig Eshhar’s T-body, the forefather of the modern scFv-based CAR receptor (140).

### Target antigen requirement

The availability and suitability of target antigens are the greatest challenges in CAR T cell therapy. Due to the high requirements for target antigens, such as high and stable expression at relevant levels and preferably overexpression of the targeted antigen on cancerous cells compared to physiological tissues (141), the selection of potential target antigens are limited (142). Furthermore, the main mode of action of CAR T cells is the recognition and signaling of the CAR receptor upon engagement with the strictly defined epitope on a target antigen (143). Thus homogenous antigen expression is important for successful CAR T cell therapy, even though low frequency of antigen heterogeneity can be addressed by so called bystander effects whereas heterogenous cancers and antigen loss require multitargeted CAR approaches (144, 145).

Bystander effects through the recruitment of immune effector cells, such as T cells, NK cells and macrophages in the tumor niche have been shown to significantly contribute to tumor control for instance in the CD19 CAR T cell trial ZUMA-1 (NCT02348216) (146) and in preclinical models (147) and shall be exploited using next generation CAR designs, engineered to inducibly express cytokines impacting on the cytokine milieu (148). These effects are relevant but shall not be overinterpreted to enable CAR T cells to successfully control antigen negative tumor lesions.

In clinical practice there are three main strategies (i-iii) pursued to balance potency and on-target-off-tumor toxicities in targeted immunotherapies utilizing antibodies, bispecific T cell engagers (BiTEs), antibody-drug conjugates (ADCs) and CAR T cells.

All strategies are characterized by the inability to target cancer specifically and thus require to appreciate the potency limit set by the on-target-off-tumor toxicities comparable to dose-limiting toxicities (149) and cumulative dose-limiting toxicities in conventional chemotherapies (150). Despite the highly antigen-specific targeting mechanism, antibodies and medicines derived thereof are targeted to surface expressed structures that are co-expressed in physiological tissues (107, 151). As a consequence, all tissues that express the targeted antigen are subject to on-target-off-tumor toxicities. The impact of this effect is defined by biological factors on the target expressing cells as well as the CAR expressing effector cells. On the target cells, these include target antigen expression level and the primary resistance or general susceptibility of the target cells to CAR mediated effects for which a first indication of tumor control with acceptable toxicity was documented using GD2- (152) and HER2-specific CAR T cells (153). On the CAR T cell side, the affinity of the recognition domain and the signaling components both define the potency and the toxicity mediated on the antigen positive cells (154).

The most successful strategy ^i)^ in CAR T cell therapy is to accept the “complete” eradication of target antigen positive cells transiently and/or permanently. This has been observed in patients treated with CD19- and BCMA-targeted CAR T cells (23, 155, 156). Importantly, the level of the depletion of physiological antigen positive cells is a strong indicator of leukemia control in B-lineage acute lymphoblastic (107) and chronic lymphocytic leukemia (157). The function of the B-lineage compartment can be substituted by immunoglobulin replacement therapy and as such displays an exception in the human body since severe tissue damages or depletion of tissues in most organs are not compatible with life. However, the strategy to eliminate specific cell subsets in the body permanently is quite unique and is reserved for CAR T cell immunotherapy in B-lineage dependent oncology (4, 92) and it has been demonstrated to be beneficial in life-threatening autoimmune diseases, such as refractory systemic lupus erythematosus (SLE) (158).

The second strategy ^(ii)^ is to transiently accept severe toxicities and then terminate the CAR T cell function after a predefined period of time. This is especially important in AML because AML-associated immunotargets are co-expressed on vitally essential myeloid bone-marrow derived cells and progenitor cells (159). CAR T cell therapy including conditioning chemotherapy with fludarabine and cyclophosphamide but especially in AML is accompanied with severe myeloid toxicities leading to an immunocompromised state with an increased risk for life-threatening infectious complications (160).

The systematic preclinical evaluation of CD33-directed CAR T cell constructs identified the CD33-targeted lintuzumab-based 2^nd^ generation CAR construct (LIN-CD28-CD3Z) to be most efficacious (161) and led to the multicenter CD33-specifc CAR T cell trial (NCT03971799) for pediatric patients with relapsed and refractory AML. In this context CAR T cells are used for remission induction prior to subsequent allogeneic stem cell transplantation. Using CD33-targeted CAR T cells requires the profound elimination of the CAR T cell function post treatment via the conditioning regimen to save patients from experiencing ongoing myelosuppression, unless patients are in parallel to the CAR T cell application, transplanted with genetically modified hematopoietic stem cells with a gene knockout for the CAR-targeted antigen, such as CD33 (162) or CD45 (163) to exclude the recovering autologous hematopoietic system from the CAR-targeting mechanism.

In the third strategy ^(iii)^ the on-target-off-tumor toxicity is tolerable and generally not life-threatening. This applies mainly to overexpressed target antigens like mesothelin (MSLN) (97), CEA cell adhesion molecule 5 (CEACAM5) (164), HER2 (153), GD2 (152), B7H3 (165), CD70 (166), IL13Ra2 (167) and others in solid cancers. The listed target antigens are expressed on a variety of different tissues or are upregulated in immune cells upon activation, such as CD70 (168, 169) and B7H3 (170). The toxicities experienced with CAR T cells for solid cancer treatment are generally tolerable at standard dose levels (152, 164) and even intracranially administered CAR T cells in brain cancers (glioblastoma multiforme) (167) are considered a legitimate approach. However, in some instances CAR T cells in solid cancer patients have been lethal due to on-target-off-tumor toxicities. In a patient with colorectal cancer, reportedly a high dose of HER2-specific CAR T cells (1x10^10^)CAR^+^ cells) induced a severe acute respiratory distress syndrome (ARDS) and subsequent cardiac arrest causing death within days (171).

One approach to overcome or limit CAR mediated on-target-off-tumor toxicities in physiological non-cancerous tissues is to reduce the affinity of the recognition domain (scFv, VHH) (172). In CD19 CAR T cells, the 40x lower recognition domain CAR induced significantly less toxicities including CRS and ICANS compared to FMC63-based CAR T cells in childhood BCP-ALL patients while demonstrating enhanced proliferative capacity and antitumor activity (108). However, reduced affinity of the recognition domain and reduced responsiveness based on the CAR architecture [4-1BB versus CD28 costimulatory domain] increases the risk for antigen low expressing tumors to evade the targeting mechanism whereas CAR persistence remains an independent discriminator of clinical success (4, 12, 108). Sparing toxicities by reducing the affinity has been demonstrated for ICAM-1 specific CAR T cells (micromolar affinity) in thyroid cancer (173), as well as in HER2- and EGFR-specific CAR T cells in various solid cancer models including ovarian and prostate (174). Genetic alteration of the CD3ζ chain signaling to reduce the number of immunoreceptor tyrosine-based activation motifs (ITAMs) alleviates exhaustion and can alter the antigen-density threshold of a CAR (175). Logic AND-gating circumvent on-target-off-tumor toxicity but in consequence lead to a sensitivity decrease of the CAR T cells since the CAR requires two independent signals for efficient CAR activation, e.g., the AND-gating of a dual-CAR construct [CEA-CD3ζ & MSLN-4-1BB] in which the CEA-CAR provides the CD3ζ signaling and the MSLN-CAR provides the 4-1BB costimulatory signaling (176).

Since allorejection limits the efficacy of CAR T cell therapies, strategies to reduce immunogenicity via de-immunization and humanization have become an integral part of CAR T design. Further, reduced immunogenicity is achieved by using fully human natural ligand- or receptor-based CAR T. By repurposing high affinity ligand-receptor interactions to target overexpressed ligands [NKG2DL via NKG2D] (177) and receptors [BCMA & TACI via APRIL] (8) on cancers have been explored in the preclinical setting and in clinical trials (178). However, the clinical success of ligand-based CAR T compared to conventional scFv-based CAR T design thus far has been disappointing in multiple myeloma (7).

### Early-onset toxicities in CAR T cell therapy

Cytokine release syndrome (CRS), Immune effector cell associated neurotoxicity syndrome (ICANS) and Macrophage Activation Syndrome (MAS) are early onset toxicities, which can have fatal symptoms (179).

The acute CRS is a common systemic inflammatory response to the excessive secretion of cytokines in the CAR T cell activation and proliferation, which occurs in 77-93% of leukemia patients treated with CAR T cells. Upon CAR T cell activation, the increased cytokine concentrations, particularly of IL6, a pleiotropic and pro-inflammatory cytokine, co-activate macrophages and monocytes, which leads to a further secretion of cytokines, and can be diagnosed from the high levels of granulocyte-macrophage colony-stimulating factor (GMCSF) in the serum. CRS is symptomized by fever, rigors, hypoxia, nausea, and heart rhythm disorders, including tachycardia and arrhythmias. In addition to heart dysfunctions, multiorgan failure can cause life-threatening medical conditions. Detections at early stages can allow for specific anti-inflammatory therapy, for instance, using tocilizumab to block the IL6 receptors, which can minimize the adverse effects (3, 180).

In the beginning of CRS, the brain is protected from primary and secondary involvement of CRS by the blood-brain barrier. Hence, the migration of CAR T cells to the brain is slower compared to other compartments of the body. However, the secretion of IL1ß and IL6 triggers von Willebrand factors, a glycoprotein, which disrupts the highly selective semi permeability of the blood-brain barrier. This permits cytokines and cells to enter the central nervous system, which changes the cytokine concentration in the brain. The elevated cytokine levels in brain tissues have been linked to neurological complications (181).

20-70% of CAR T cell patients develop neurological symptoms, including initial manifestations of cognitive impairment, such as tremor, confusion, lethargy, mild expressive and receptive aphasia, stupor, apraxia and decreased attention, which can progress into severe ICANS, symptomized by agitation, status epilepticus, cerebral oedema and occasionally intracerebral hemorrhage (3, 182, 183).

In CD19-targeted CAR T cells, the primary on-target-off-tumor toxicity occurs in the B-lineage tissues due to the expression of CD19, a BCR co-receptor on B cells. However, due to low expressions of CD19 in neural cells, on-target-off-tumor toxicities may also occur in neural tissue in the hyperinflammatory state. Furthermore, there are associations between the serum elevations of cytokine IL15, IL3 and GM-CSF and the blood-brain barrier permeability and ICANS development and severity (181, 184). Nonetheless, the fundamental principles, responsible for the neural toxicities are not thoroughly comprehended and other factors are considered to influence the severity of ICANS symptoms.

Hemophagocytic lymphohistiocytosis (HLH) and macrophage activation syndrome (MAS) are severe hyperinflammatory syndromes, associated with the pathological dysregulation of macrophage proliferation, which are mostly triggered by viral infections, especially Epstein-Barr virus infection, genetic lymphoid immune cell disorder, rheumatic autoimmune diseases, for instance the systemic-onset juvenile idiopathic arthritis, systemic lupus erythematosus and genetic mutations (3, 185–187). In addition, HLH and MAS can occur in the context of BiTE and CAR T cell immunotherapy (185). The excessive release of proinflammatory cytokines IL1, IL6, IL18 and tumor necrosis factor, triggered by the uncontrolled activation and proliferation of macrophages, leads to dysregulated immune activity and induces systemic inflammation (186). MAS is characterized by hyperinflammatory clinical features, including persistent fever, cytopenia, coagulopathy, liver insufficiency, hepatosplenomegaly, lymphadenopathy and multi-organ failure (3, 186). Laboratory diagnostic characteristics are a decrease in blood cell count due to hemophagocytosis, hyperferritinemia, abnormal coagulation profile, elevated triglycerides and high cytokine levels. The treatment of MAS usually involves immunosuppressive medications, such as corticosteroid and immunomodulatory drugs, for instance IL1 and IL6 signaling inhibitors (179, 181).

### Immunosuppressive tumor microenvironment

A significant constraint in the effectiveness of CAR T therapies, particularly in treating solid tumors, is the immunosuppressive nature of the tumor microenvironment (TME) (188). The TME is composed of different cell types, matrix proteins and secreted factors (189). Immunosuppressive cell types like myeloid-derived suppressor cells (MDSCs), tumor-associated macrophages (TAMs), and regulatory T cells (Tregs) produce anti-inflammatory cytokines, such as interleukin-10 (IL10), IL4, transforming growth factor-beta (TGF-β) that inhibit the CAR T function. These cytokines hinder CAR T function, impede proliferation, induce T-cell exhaustion, reducing their efficacy against tumor cells by limiting their cytotoxic and cytokine-producing abilities. Additionally, dense fibrogenic extracellular matrix, dysregulated tumor vasculature, and the hypoxic TME, characterized by limited nutrients and oxygen, restrict CAR T infiltration and function (188).

Efforts to enhance CAR T efficacy involve engineering these cells to resist the TME-induced exhaustion or express additional effector functions to promote persistence and functionality within the immunosuppressive TME.

One strategy to overcome exhaustion is to target T cell intrinsic pathways, such as programmed death-1 (PD-1) or TGF-β (190). Activation of the TGF-β receptor upon binding of TGF-β present in the TME upregulates CD70 expression and induce T-cell exhaustion with increased expression of the inhibitory receptors PD-1 and TIM-3 (191). Sustained PD-1 expression positively correlates with an exhausted phenotype and dysfunction of the T cells (192, 193). Pre-clinical data indicates that knocking out of PD-1 and/or TGF-β receptor II genes in CAR T cells with CRISPR/Cas9 technology improve the CAR T function against tumors expressing PD-L1 and/or TGF-β release (194, 195). Current clinical trials, evaluate anti-MUC1 CAR T cells with PD-1 knockout in advanced esophageal cancer (NCT03706326) and anti-EGFR CAR T cells with TGFBR2 knockout in EGFR-positive solid tumors (NCT04976218). Expression of dominant-negative PD-1 or TGF-β receptors, receptors lacking intracellular domains necessary for downstream signaling, also show promising improvement of the CAR T persistence and have been shown to be safe in a phase I clinical trial (196, 197). Combining CAR T therapy with checkpoint inhibitors with already approved immunotherapies, such as anti-PD-L1 or CAR T cells engineered to secrete PD-1 scFv or nanobodies, promote the anti-tumor activity (97, 198). Other approaches such as chimeric switch receptor-expressing CAR T cells like PD-1/CD28 or TGF-β/IL7 also seems to improve the persistence of CAR T (199, 200).

Another strategy to enhance the anti-cancer functions of CAR T cells and recruit resident immune cells by modulating the TME is to use CAR T cells designed to produce pro-inflammatory cytokines (201). Many different cytokines have been used to potentiate CAR T cell function, such as interleukin (IL) IL7 (202), IL15 (134), IL21 (203), IL12 (204) and IL18 (86). Anticancer activity is substantially induced by the highly potent pro-inflammatory cytokine IL12, which is naturally secreted by monocytic cells, e.g., dendritic cells and macrophages. IL12 enhances the cytotoxic capacity of both NK cells and T cells by inducing granzyme B and perforin production and interferon (IFN)-γ secretion (205, 206) and reduce the immunosuppressive effects of regulatory T cells (207). However, despite the tempting and promising enhanced anticancer function of pro-inflammatory cytokine support for CAR T cells and accessory immune effector cells, severe life-threatening toxicities limit their use and require the incorporation of advanced safety measures (208). The use of inducible promoters incorporating responsive elements of NFAT, AP-1, NFκB, or other transcription factors and their combinations facilitates activation-related gene expression (209–211). Alternatively, small molecule-regulated gene expression systems, such as the tetracycline-dependent Tet-On system, can improve the control of transgenes, including CAR expression (212) and cytokine secretion. While gene regulation can be complex and prone to leakage, site-specific integration of transgenes offers new opportunities to harness physiological gene regulation mechanisms (57, 110). This approach can reverse immune functions, such as integrating activating transgenes (e.g., CAR) into inhibitory loci like immune checkpoint receptors (213). Functional control of transgenes at the protein level is more reliable (214).

## Clinical experience with adapter CAR technologies

Various adapter CAR T cell systems are being evaluated in clinical trials for their efficacy and safety in treating different types of cancers. Although these technologies utilize the fundamental principle of adapter CAR technology, each employs a unique adapter molecule format with differential size and pharmacokinetic and pharmacodynamic properties, including scFv (78, 215), monomeric Fab (79), silenced full-size antibodies (216), and d-domains (80). Additionally, the adapter molecule dosing can be significantly lower (10-1,000 times) than traditional antibody dosing used for therapeutic IgG1 antibodies like rituximab (217) at 375 mg/m^2^ and cetuximab (218) at 400 mg/m^2^ for most technologies, with exemption of the high-affinity CD16-polymorphism (158V) (73).

In the clinical trial ATTCK-20-03 (NCT03189836), the CD16-derived antibody-coupled T cell receptor (ACTR707) product was used in conjunction with rituximab at 375 mg/m² for the treatment of relapsed or refractory CD20+ B-cell lymphoma. Patients achieved a complete remission rate of 50% (3/6) with an acceptable toxicity profile (219). In the follow-up, 56% (14/25) of patients responded, with 40% (10/25) achieving complete remission. Although the trial was discontinued due to the adverse event of neutropenia, it serves as a proof-of-concept for ACTR-based CAR therapy (220).

The peptide-based sCAR system (derived from the yeast GCN4 transcription factor) is currently being evaluated in patients with relapsed/refractory B-cell Malignancies (NCT04450069). The first-in-human data involved two patients with follicular lymphoma and one patient with mantle cell lymphoma, who received 140x10^6^ CAR^+^ cells in conjunction with 10 µg/kgBW/day SWI019, a CD19-targeted monomeric Fab fragment used as adapter molecule. Two patients achieved a complete response according to Lugano criteria. Acute toxicity, ICANS was successfully managed with dexamethasone and by reducing the SWI019 dose to 5 µg/kgBW (79). Another peptide-based adapter CAR system (UniCAR, derived from the nuclear autoantigen La/SS-B) is being clinically tested in patients with hematological malignancies (NCT04230265). The first patients treated with relapsed/refractory AML (rrAML) received 100 or 250x10^6^ CAR^+^ cells with 0.5 or 1 mg/day TM123, a CD123-targeted scFv-based adapter molecule. Complete hematological remission with incomplete recovery was observed (215). In a follow-up report, 19 patients showed an overall response rate (ORR) of 53% (8/15) for rrAML and 75% (3/4) in patients with AML at minimal residual disease (MRD) level (78). The adapter CAR technology IBI345, utilizing silenced antibodies carrying the P329G substitution, is used for the treatment of claudin 18.2 (CLDN18.2) positive tumors, including esophagogastric junction-, gastric-, and pancreatic cancer (NCT05199519). Patients were administered 50-250x10^6^ CAR^+^ cells plus 1 mg/kgBW/day IgG1, demonstrating limited clinical efficacy (216). Another promising technology in clinical evaluation is the d-domain-based adapter CAR system (SparX) for the treatment of multiple myeloma (NCT04155749) and AML (NCT05457010). The adapter molecules are d-domain-based and targeted to BCMA in multiple myeloma and CD123 in AML (80). Regarding increased safety compared to conventional CAR technologies, the sCAR and UniCAR trials have demonstrated clinical proof-of-concept for reducing toxicity by pausing the administration of adapter molecules (78, 79, 215).

## The CAR configurator

The concept of universal CAR technologies is fascinating and tempting. Still in the early stages, adapter CAR technologies have shown first indications of clinical efficacy in humans, reported in 2021 in the clinical trial (NCT04230265) utilizing the UniCAR in conjunction with a CD123-targeted adapter molecule in relapsed/refractory AML (215). Furthermore, there are several clinical trials recruiting patients using an alternative peptide-tag based CAR system sCAR (switchable CAR) with CD19-targeted adapter molecules (NCT04450069) as well as a d-domain based BCMA-targeted trial in multiple myeloma (NCT04155749). As of today, no clinical outcomes of the sCAR and d-domain CAR trials have been published. Additionally, third-party CD19-CAR (21) and recently “first-ever” iPSC-derived CD19-CAR T cells have demonstrated preliminary clinical anticancer activity (221).

Nonetheless, since multitargeting is widely accepted the key challenge to address tumor antigen heterogeneity and antigen loss, adapter CAR technologies hold the promise to overcome this very limitation of contemporary CAR T design. Even though CD19-CD22 dual-targeted CAR constructs incorporated into one immune receptor (144) have not yet clearly proven to significantly increase long-term survival in patients, the future aspiration is that multitargeted approaches utilizing dual- or trispecific CAR products will prevent immune evasion (144, 222). Hence, versatile multitargeted adapter CAR technologies represent a pivotal stride towards the future of cancer immunotherapy. These innovative technologies have the potential to revolutionize treatment approaches by enabling the customization of patient-specific regimens, intricately tailored to the distinct antigen immune profiles of individual patients.

Besides finding the most suitable combination of targetable antigens, CAR T design will be most efficacious if genetically adjusted to the immune requirements of the specific tumor and combined with complementary treatment strategies. The complementary use of existing medicines can instantly contribute to improved outcomes. For instance, CAR T cell therapy in conjunction with immune checkpoint inhibitors, such as pembrolizumab has demonstrated to reverse CD19-CAR exhaustion in B-cell lymphoma patients (223) and enhance anticancer activity in malignant pleural disease using MSLN-targeted CAR T (97). Further, small molecules (224) and the application of cytokine or chemokine modulating chemotherapy (225) can improve the clinical efficacy of CAR T cell therapies.

**Figure 3:**
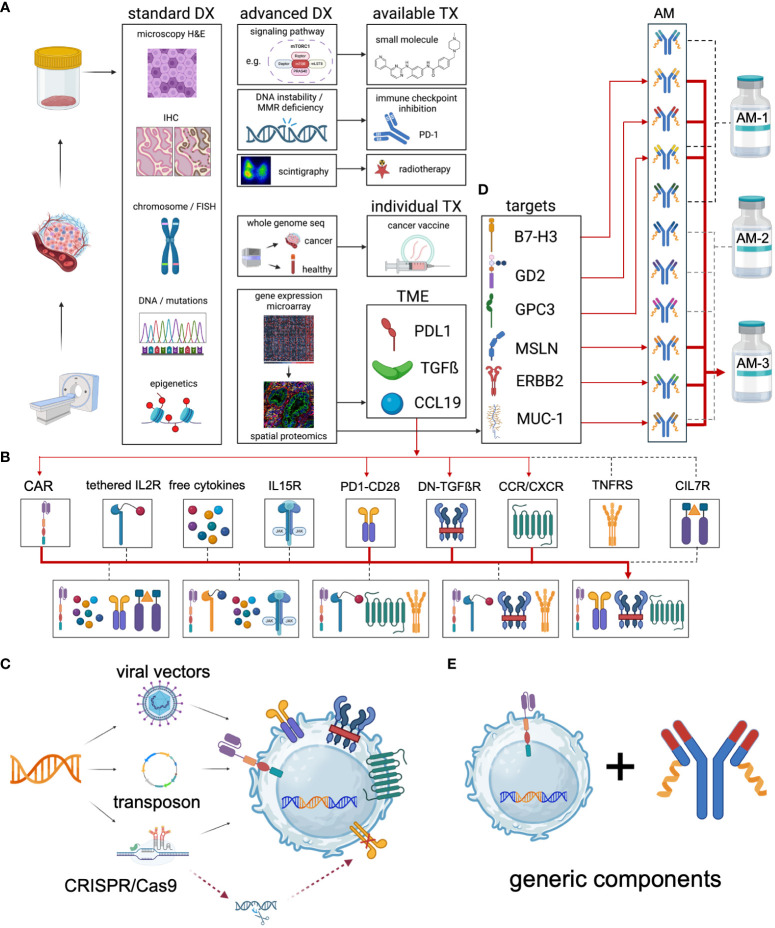
Universal CAR configurator. The concept of the Universal CAR Configurator is to design the most effective individual universal CAR for each specific cancer indication. It can be used with autologous immune effector cells or more advanced with iPSC-derived cell products to create “Universal CAR 2.0”. **(A)** The process begins with a comprehensive analysis of the cancer using state-of-the-art diagnostics. This includes basic microscopy, immunohistochemistry, and the detection of chromosomal aberrations (both structural and numerical) as well as utilizing fluorescence *in situ* hybridization (FISH). Additionally, targetable mutations and epigenetic changes are identified. Standard diagnostics confirm the diagnosis and guide the initial treatment regimen. Advanced diagnostics are employed to identify patients who could benefit from additional treatment options, either through established therapies or personalized treatments. Established therapies may involve the use of small molecules to inhibit upregulated signaling pathways such as kinases, mTOR, ABL, c-KIT, and FLT3. Moreover, patients with DNA instability due to mismatch repair deficiency, such as those with colorectal cancer, can significantly benefit from immune checkpoint inhibition (226). Further, theranostic approaches with antibody-guided radionuclides, such as Iodine-131 coupled to antibodies are used therapeutically (227). Whole genome sequencing is used to identify targetable mutations in the tumor with patient-individual cancer vaccines (228). Functionally tailored CAR-based therapies require a patient-individual two-step immune profiling. In the first step, the cancer is screened for the expression of relevant genes. Based on the results, in a second step a specific antibody panel is used for spatial proteomics to elucidate the patient’s tumor microenvironment (TME), exemplified as 1^st^ PDL1 expression, 2^nd^ TGFß secretion and 3^rd^ CCL19 chemokine release by the tumor. **(B)** Therefore, the design of the TME-adjusted patient-individual CAR product requires the incorporation of 1^st^ a PD1-CD28 signal converting receptor, 2^nd^ a dominant negative TGFß receptor and 3^rd^ the introduction of the chemokine receptor CCR7 to support the enrichment of CAR effector cells in and around the tumor. **(C)** The genetic payload of the relevant additional effector functions may be incorporated into the expression cassette and transferred via stable integration of the transgene using viral, transposon-based or CRISPR-based gene delivery. Additional genetic modifications can be utilized to inactivate immunosuppressive receptors such as PD-1 or CTLA-4 and others. **(D)** Spatial proteomics is used for the selection of patient-individual antibody panels that are specifically formulated for each patient. Combinatorial targeting is the key obstacle of targeted immunotherapies to overcome the antigen heterogeneity and immune escape evasion mechanisms. **(E)** Although patients receive individualized therapies, they built on generic key components - the CAR receptor and the adapter molecules. DX, diagnostics. TX, therapeutics. AM, adapter molecule. TGFß, TGF beta. IL15R, IL15 receptor. PD1-CD28, signal converting receptor. DN-TGFßR, dominant negative TGF beta receptor. CCR/CXCR, chemokine receptors. TNFRS, tumor necrosis factor receptor superfamily. CIL7, constitutive IL7 receptor.

As indicated in **Figure 3** Universal CAR configurator, the comprehensive tumor profiling will facilitate to identify the best suitable complimentary therapies and help select the potential immune targets for a patient-individualized adapter molecule panel. Most importantly, it will provide the objective which additional effector functions may specifically increase the CAR performance in the corresponding subject.

These additional genetic modifications shall exploit pathways supportive of anticancer functions, such as cytokine signaling (IL7, IL12, IL15, IL18, IL21 and others) (86, 200, 204) and activating co-stimulatory receptor signaling (CD28, OX40 and others) (199) as well as the homing preference of cells via provision of chemokine receptors (229). Furthermore, they shall inactivate negative regulators of CAR T cell function, such as TGFßR signaling and immune checkpoints to overcome the immunosuppressive tumor microenvironment (TME) (195, 198, 200, 230). The disruption of immune homeostasis can lead to life-threatening, uncontrollable detrimental toxicities. Thus, in conventional CAR design it is more critical to employ a combination of boosting effector functions that may cause unmanageable hyperinflammatory complications if the targeted antigen is broadly expressed in physiological tissues. The decoupling of antigen recognition and signaling in adapter CAR technologies alone facilitates to control the unleashed CARs in the patient (20, 65). In summary, the development of effective CAR immunotherapies requires decoding the immunological processes in the tumor and converting them into effective and safe additional effector functions.

On the adapter molecule side, pharmacokinetic and pharmacodynamic variables need to be implemented to ensure high functionality of the adapter CAR system in all body compartments. Besides the critical antigen density (12), other factors like antigen shedding (231), antigen turnover and internalization kinetics (232) require the mindful selection of adapter molecule formats. They shall reach all various immune compartments. To overcome the blood-brain-barrier, adapter molecules either must be applied into the central nervous system (CNS) which is a viable administration route (233, 234) or be able to penetrate into the CNS (235). Certainly, they need to reach sufficient concentrations in the primary tumor niche and in metastases (236).

**Figure 4:**
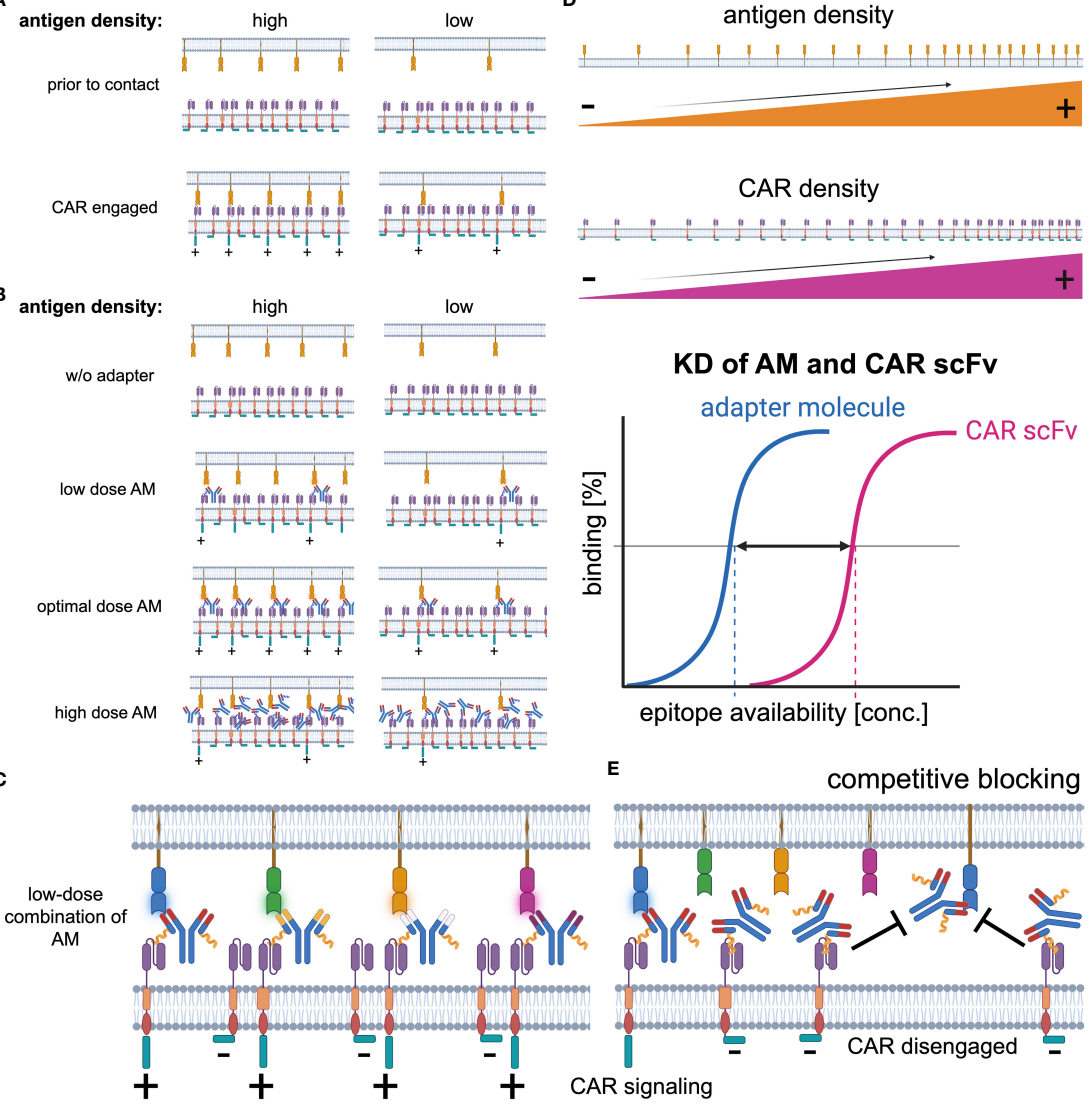
Key determinants of CAR signaling. **(A)** The CAR receptor-target antigen interaction in conventional CAR technologies is primarily determined by the antigen expression. Most target antigens are expressed at lower levels than the CAR receptor on the effector cell population. Thus, in antigen high expressing target cells, the avidity is higher than in antigen low expressing targets and as the CAR signaling is proportional to the CAR receptor-target antigen interaction, it is enhanced in antigen high expressing targets. **(B)** Besides the target antigen density, in adapter CAR technologies the adapter molecule concentration significantly impacts on the CAR engagement with the target cells. Without any adapter molecule available, there is no CAR engagement possible. With increasing concentrations, the CAR engagement becomes more efficient and reaches an optimum before inhibitory effects start to reduce the CAR-target interaction at supra-optimal adapter molecule concentrations. **(C)** Synchronic multitargeting, utilizing a combination of adapter molecules at low concentrations, can increase the CAR-target interaction while the blocking effects are reduced. **(D)** Key determinants of CAR-target interaction are ^i)^ the antigen expression density on the target cells, ^ii)^ the CAR receptor expression density on the CAR^+^ effector cell population, and the ^iii)^ interplay of the avidity and the affinity (K_D_) of the adapter molecule to the targeted antigen as well as the CAR recognition domain, e.g., CAR-scFv to the CAR-target moiety on the adapter molecules. **(E)** At supra-optimal concentrations, competitive blocking effects reduce the probability of CAR-target interaction and therefore pharmacokinetic as well as pharmacodynamic aspects must be considered optimizing the adapter molecule dosing.

By nature, adapter CAR T will always have a lower avidity to the targeted antigen than conventional CARs, illustrated and outlined in **Figure 4**. However, multitargeted strategies and performance tuning can compensate for the reduced initial formation of the cytolytic synapse and turn the disadvantage into dominance. Additionally, adapter CAR T cell technologies facilitate a more physiological CAR recruitment and engagement compared to conventional CAR design and allow cells to recover from the massive CAR stimulation according to the administration regimen of the adapter molecules (237).

## Specific limitations and challenges of iPSC-derived adapter CAR therapy

While there are numerous advantages of iPSC-derived adapter CAR products, summarized in **Table 2**, there are limitations and challenges in the creation of universal (iPSC-derived) adapter CAR effector cells, referred to as universal CAR 2.0.

**Table 2:** Features of universal CAR products.

iPSC-derived CAR product	*Characteristics*	*Consequence*
*Critical aspects*		
Manufacturing time	off-the-shelf product	available at any time
Manufacturing costs	off-the-shelf product	cost reduction
Manufacturability	off-the-shelf product	unlimited supply
	off-the-shelf product	standardized and consistent
Safety profile	thorough characterization	enhanced safety
Efficacy	selection for potency	enhanced potency
Customized products	designed for specific purpose	enhanced potency and safety
Ethical considerations	iPSC derived from adult cells	bypassing ethical concerns of embryonic stem cells
Adapter CAR product		
*Critical aspects*	*Characteristics*	*Consequence*
Manufacturing	generic CAR	cost reduction
	generic adapter molecule	cost reduction
Acute toxicities (CRS, ICANS, MAS)	switch-on mechanism	improved safety profile
On-target-off- tumor toxicities	switch-on mechanism	transient toxicity
	sequential targeting	distribution of toxicity
	treatment in cycles	reconstitution of tissues
Antigen loss, immune evasion	combinatorial multitargeting	improved performance
Antigen loss, immune evasion	sequential multitargeting	improved performance
Temporally limited CAR recruitment	treatment in cycles	reconstitution of effector cell function
Customized products	designed for specific purpose	patient-individualized combinatorial targeting

The table highlights the features of universal CAR 2.0, showcasing its next-generation design that integrates additional effector functions and enhanced safety measures. It also outlines the benefits of iPSC-derived CAR products in manufacturing and the advantages of adapter CAR products, such as improved safety and efficacy through on-switch and combinatorial targeting. This unified cellular product enables patient-specific therapy tailored to the target antigen expression profile of each cancer patient. iPSC, induced pluripotent stem cells; CRS, cytokine release syndrome; ICANS, immune effector cell-associated neurotoxicity syndrome; MAS, macrophage activation syndrome.

A significant current challenge with the use of artificially engineered third-party effector cells is their notably shorter lifespan compared to autologous CAR therapies (238). Despite sophisticated design efforts, there remains a substantial gap in our understanding of the potential long-term risks associated with these therapies. Concerns particularly focus on the possibility of malignant transformations due to unintended genetic modifications and contamination with undifferentiated stem cells (239), as well as autoimmune or hyperinflammatory reactions. These adverse effects may arise from specific combinations of genetic alterations that promote inflammatory responses via activating receptors and cytokines while simultaneously deactivating inhibitory receptors, e.g. PD-1, disrupting the immune system’s natural regulatory balance (240).

The primary challenge of the adapter CAR function is largely influenced by the biodistribution of adapter molecules and their ability to penetrate less vascularized and perfused body compartments, such as the testicles, the central nervous system (CNS), and particularly fibrotic and encapsulated solid tumors (241). Additionally, the pharmacokinetics and compatibility of specific adapter molecules can complicate the design and outcomes of clinical trials. It is improbable that a uniform adapter molecule format will be effective across different types of cancers. Consequently, in clinical trials, the format of the adapter molecule will dictate the administration route and regimen. Furthermore, combinatorial targeting will necessitate intricate protocols and meticulous monitoring during the optimization of treatment regimens.

## The bottom line for successful CAR therapy

CAR products are designed to equip immune cells with novel effector functions and combine these with their natural capacity for adaptation to everchanging circumstances. These multifaceted adaptive functions allow the CARs to migrate efficiently to any part of the body via chemotaxis (242), then to mediate a potent but considerate immune response that does not lead to detrimental effects and unrepairable tissue damage (243). The immune response is complemented by the recruitment of accessory immune cells via the secretion of proinflammatory and immunosuppressive cytokines, chemokines as well as the presentation of immunomodulatory ligands to shape and orchestrate the immune response (244). During the acute phase of the response the vertical differentiation and exponential multiplication of the effector cells is imminently important (4). However, to maintain a strong immune response the horizontal proliferation (maintenance), differentiation and maturation of immune cells provide the ability for self-renewal and long-lived immune memory function (245) which are the prerequisite for the comprehensive elimination of cancer and subsequent protection from recurrence (4).

In consequence, the most promising effector cells of the future are a reflection of the complex natural cellular immunity (246) in artificially designed effector cells that are equipped with versatile synthetic receptors (57, 110, 175, 247). Therefore, the appreciation and incorporation of natural, multilayered immune functions will lead to the most advanced cellular products equipped with the versatile biological plasticity, capable of autonomously regulated adaptability, self-renewal, and the persistence of immune memory (245). Long-lived immune memory allows resting memory T cells to awaken and protect humans from harm (virus, bacteria, fungi and cancer) after re-encounter with invaders even with the latency of many years and displays one of the most impressive evolutionary immune functions (114).

All of the named immune functions have only been partially understood but have guaranteed immune protection and thus certainly shall be considered in the design of novel cell products. The practical challenge in creating cellular medicines is to retain these features while adding beneficial effector functions which is supported by shorter manufacturing protocols (248).

Presently, the scope for clinical applications is confined to meticulously defined cellular products prevailing from the legislative landscape and the regulatory authorities’ inclination towards stringent product specifications (249). The encounter with cellular therapies has underscored the intricacies within the immune compartment. Yet, the remarkable diversity exhibited by T cells stands as a valuable asset in tackling immunological challenges. This resilience has been notably exemplified in the realm of CAR T cells, laying the groundwork for future explorations in the design of next-generation CARs.

In conclusion, universal allogeneic CAR T cell products hold the promise to overcome the economic hurdles of personalized cellular medicines with regards to infrastructure, logistics as well as manufacturing and distribution costs (250). Additionally, they support to broaden the applicability of CAR T cell therapy in patients that do not meet the basic CAR manufacturing requirements, e.g., reduced general condition, low lymphocyte count, poor immune status or simply lack the time required for the manufacturing of autologous CAR T cell products before initiating the treatment (28). Whilst allogeneic universal iPSC-derived cellular therapies are promising, they reside at a very early stage of development and are mainly compromised by allorejection that limits persistence and their overall potency of the anticancer response (251). In the coming years, preclinical and clinical research will need to provide more insight into both safety and efficacy of iPSC-derived CAR products to improve this novel, potentially cost-effective and life-saving technology (28, 251) that may become a complementary treatment to “conventional CD19- and BCMA-specific CAR T cell therapies” in the future (4, 23, 101).

Numerous adapter CAR technologies have been developed with the aim to introduce a safety on-switch to control early onset toxicities and on-target-off-tumor toxicities as well as to establish versatile patient-individualized combinatorial immunotargeting (20, 65). Incorporating next-generation CAR design (209) may catapult adapter CAR technologies to outcompete conventional CAR designs while retaining safety (237). The fusion of these distinct two universal CAR technologies unites key features of two concepts that might resolve our current skepticism for clinical potency and safety as well as the significant costs required for the development. In summary, groundbreaking advancements remain pivotal to usher in this new era of two-component universal CAR cellular therapies, based on bespoke patient-individualized treatments, derived from readily available off-the-shelf products.

## Author contributions

LS: Conceptualization, Validation, Visualization, Writing – original draft, Writing – review & editing. CW: Writing – original draft, Writing – review & editing. MB: Writing – original draft, Writing – review & editing, Resources. PS: Conceptualization, Formal analysis, Funding acquisition, Project administration, Resources, Supervision, Validation, Visualization, Writing – original draft, Writing – review & editing.
